# Phylogeographic studies of schizothoracine fishes on the central Qinghai-Tibet Plateau reveal the highest known glacial microrefugia

**DOI:** 10.1038/s41598-017-11198-w

**Published:** 2017-09-08

**Authors:** Yangyang Liang, Dekui He, Yintao Jia, Heying Sun, Yifeng Chen

**Affiliations:** 1 The Key Laboratory of Aquatic Biodiversity and Conservation of Chinese Academy of Sciences, Institute of Hydrobiology, Chinese Academy of Sciences, Wuhan, 430072 China; 2 Southeast Asia Biodiversity Research Institute, Chinese Academy of Sciences, Menglun, Yunnan, 666303 China; 3 University of Chinese Academy of Sciences, Beijing, 100049 China

## Abstract

Pleistocene climatic oscillations have greatly influenced the evolutionary history and distribution pattern of most extant species. However, their effects on species on the Qinghai-Tibet Plateau (QTP) are not well understood. To investigate the effects of past climatic shifts, particularly the Last Glacial Maximum (LGM), on plateau fish, we analysed the phylogeographic structure and demographic history of five closely related taxa of the subfamily Schizothoracinae, a representative endemic taxon of the QTP, from nine endorheic lakes on the central QTP and three peripheral exorheic rivers using the mitochondrial control region (D-loop) sequence and 12 microsatellite (SSR) markers. Phylogram from D-loop haplotypes revealed two well-supported lineages (North and South) separated by the Tanggula Mountains. The results from the D-loop and SSR revealed that endorheic populations possess high genetic diversity and a unique genetic structure. The most recent demographic expansion occurred post-LGM for most endorheic populations and in the last interglacial period for Siling Co and all exorheic populations. Phylogeographic structure, together with species distribution modelling, supports the scenario of multiple glacial refugia on the QTP during the LGM and suggests that Siling Co (4540 m asl) is a cryptic glacial microrefugia for plateau fish, which would be the highest glacial microrefugia known.

## Introduction

The advances and retreats of glaciations through the Pleistocene, especially most recently the Last Glacial Maximum (LGM, 19–26 thousand years ago, kya), have significantly affected the demographic history, abundance and distribution pattern of modern species^1–3^. During these glaciations, many species were excluded from large parts of their ranges and forced into refugia by the expanding ice sheets, followed by recolonization and population expansion as the glaciers retreated^1^. For example, in Europe, palaeontological records and genetic analyses have revealed that many species were retreated southward to the three Mediterranean peninsulas (Balkans, Italian and Iberian) during the LGM and expanded northward into their current distribution areas in the subsequent warming period^2, 3^. Recently, biogeographic studies have led to the widespread acceptance of northern extra-Mediterranean refugia from the Atlantic coast of France in the west to the Black Sea region in the east, for example, the Carpathian Basin and even northern Central Europe^4^. Using phylogeographic studies, increasing numbers of previously unknown, or cryptic, refugia are identified^3, 5^.

The Qinghai-Tibetan Plateau (QTP) is the world’s highest and largest plateau, covering an area of 2.5 million km^2^ and has an average altitude of 4500 m above sea level (asl). The QTP and adjacent mountains are the largest glaciated tracks outside the polar region^6^. Both plateau uplifting and Pleistocene climatic oscillations have greatly influenced the biotic pattern on the QTP^7, 8^. Although the uplifting progress of the QTP remains controversial, most studies agree that the QTP attained its current state at least 150 kya before the last interglacial period (130–25 kya)^7, 9^. The present biotic pattern on the QTP was mainly shaped by Pleistocene climatic oscillations^6^. Unlike Europe and North America, where continental glaciations occurred, only mountain and valley glaciers formed on the QTP during the LGM^10, 11^. Therefore, in the common “edge refugia” model, the low-altitude southern and eastern edge of the QTP served as the glacial refugia for the plateau species; this model has proved insufficient for a comprehensive understanding of the effects of glaciations on the biota of the QTP^12–14^.

The influence of Pleistocene glaciations on the QTP have been studied in various species, but several basic questions, such as the locations of glacial refugia and the process of postglacial colonization, remain poorly understood^13–15^. Some studies of endemic birds and plants have suggested that the south-eastern edge of the QTP acts as the glacial refugia^15–18^, while others have failed to detect “edge refugia” but suggest multiple refugia on the platform of the QTP^12, 19, 20^. Correspondingly, two main scenarios for the biota on the QTP have been proposed, *i.e*. postglacial recolonization from peripheral refugia or the survival of the LGM *in situ*, with several refugia on the platform. For both scenarios, evidence for the location of refugia and postglacial colonizing routes is scant^13, 15^. Previous studies have mainly focused on plants, birds and amphibians in the periphery of the QTP. Therefore, more studies about additional endemic species on the central QTP are necessary for a thorough understanding of the influence of Pleistocene glaciations on the QTP biota.

Freshwater fishes are regarded as the ideal subjects to explore the effects of glaciations because of their restriction in glacial refugia that are peripheral to ice sheets and the restrictive aquatic requirements for postglacial dispersal^21^. Schizothoracine fishes (Teleostei: Cyprinidae) account for the largest component of the QTP ichthyofauna and are widely distributed in local lakes and rivers^22, 23^. They have evolved a number of adaptations to cold-water environments and are regarded as the most diverse vertebrates on the central QTP^24, 25^. Many lakes are located on the central QTP, and most are endorheic lakes^26^. As the migratory range of fish was limited by watercourses, it is likely that fishes in central QTP lakes recolonized postglacially from local refugia rather than “edge refugia”. Therefore, these lakes are ideal sites for investigating the potential of the central QTP to harbour glacial refugia.

In this study, we analysed the phylogeographic structure and demographic history of schizothoracine fishes on the central QTP and their closely related species in peripheral exorheic rivers using mitochondrial DNA (mtDNA) control region (D-loop) sequence and nuclear microsatellite markers (SSR). Species distribution modelling (SDM) was also used to predict the suitable habitat ranges for these schizothoracine fishes during the LGM. We sought to (i) assess the genetic diversity, phylogeographic structure and demographic history of those populations; (ii) infer whether the present schizothoracine fishes in the central QTP endorheic waters were recolonized from low-altitude peripheral rivers or persisted on the plateau platform through the LGM; and (iii) determine whether cryptic glacial refugia on the central QTP exist.

## Results

### Sequence characteristics and population genetic diversity

**Figure 1:**
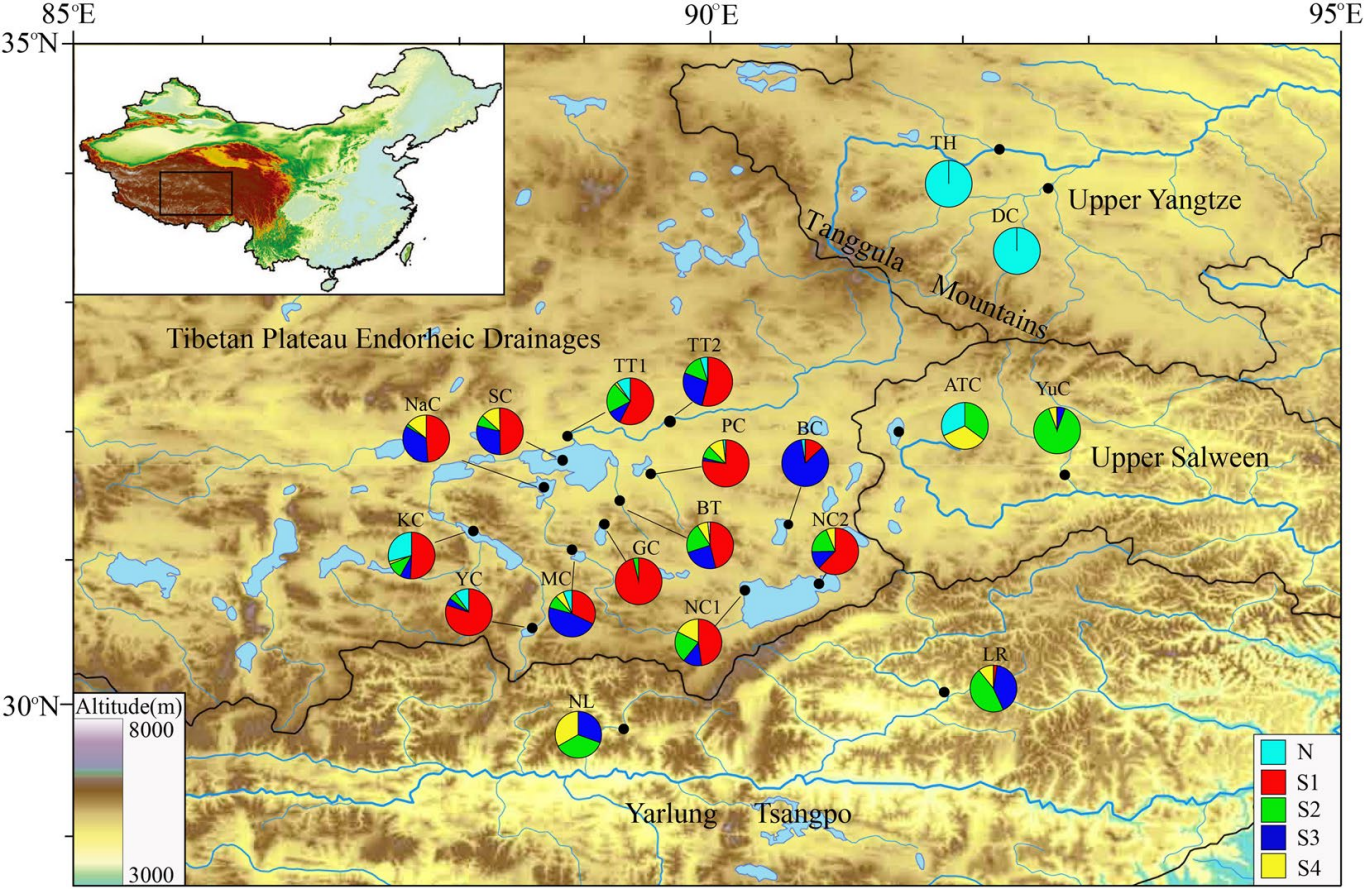
Location and haploclade composition of each sampling site in this study. Pie charts exhibit the frequency of lineages or sublineages in each population. Five colours (cyan, red, green, blue and yellow) correspond to one North lineage (N1) and four South sublineages (S1, S2, S3 and S4) in the D-loop phylogram, respectively. The boundaries of the main drainage basins are also shown by black lines. Whole names for each site are listed in Table 1. The map was drawn using ArcGIS v10.0 and Adobe Illustrator CS5 v15.1.0.

We sampled the only two taxa of schizothoracine fishes (*Gymnocypris namensis* and *Schizopygopsis* spp.) in nine endorheic lakes on the east Changtang Plateau, which is the central part and the earliest uplifted block of the QTP^27^, as well as their closely related species (*Schizopygopsis younghusbandi*, *Schizopygopsis thermalis* and *Herzensteinia microcephalus*), in peripheral exorheic rivers (upper Salween, Yangtze River and Yarlung Tsangpo). A total of 839 specimens belonging to five closely related taxa were collected from 19 sites in 2010 and 2015, ranging from 23–89 individuals per site (Fig. 1 and Table 1).

**Table 1:** Sample size and geological information of each site in this study.

Species	Site	Code	Drainages	Location	Altitude(m asl)	N(m)	N(n)
SS	Tsachu Tsangpo1	TT1	Siling Co	32°03′N 89°08′E	4561	53	30
	Tsachu Tsangpo 2	TT2		32°24′N 90°44′E	4710	57	30
	Siling Co	SC		31°47′N 88°48′E	4540	38	37
	Bochu Tsangpo	BT		31°34′N 89°19′E	4608	57	55
	Nagtsang Co	NaC		31°36′N 88°41′E	4568	49	41
	Mokiu Co	MC		31°03′N 89°01′E	4680	34	34
	Kyaring Co	KC		31°16′N 88°06′E	4654	55	52
	Yueqia Co	YC		30°28′N 88°37′E	4810	41	41
	Pongok Co	PC	Pongok Co	31°41′N 89°36′E	4545	58	50
	Gomang Co	GC	Gomang Co	31°15′N 89°13′E	4636	26	26
	Bam Co	BC	Bam Co	31°22′N 90°33′E	4565	38	35
GN	Nam Co1	NC1	Nam Co	30°55′N 90°56′E	4729	23	23
	Nam Co2	NC2		30°55′N 90°56′E	4729	32	31
SY	Namulin	NL	Yarlung Tsangpo	29°41′N 89°05′E	4005	34	30
	Lhasa River	LR		31°11′N 91°20′E	4048	32	30
ST	Amdo Tsonak Co	ATC	Upper Salween	31°56′N 91°29′E	4599	89	30
	Yu Chu	YuC		31°12′N 91°48′E	4704	56	30
HM	Dri Chu	DC	Upper Yangzte	33°52′N 92°22′E	4569	31	31
	Tuotuo He	TH		34°13′N 92°26′E	4542	36	35

SS = *S.Spp*, GN = *G. namensis*, SY = *S. younghusbandi*, ST = *S. thermalis*, HM = *H. microcephalus*, N(m), number of specimens used for D-loop sequence analysis, N(n), number of specimens used for nuclear microsatellite analysis.

**Figure 2:**
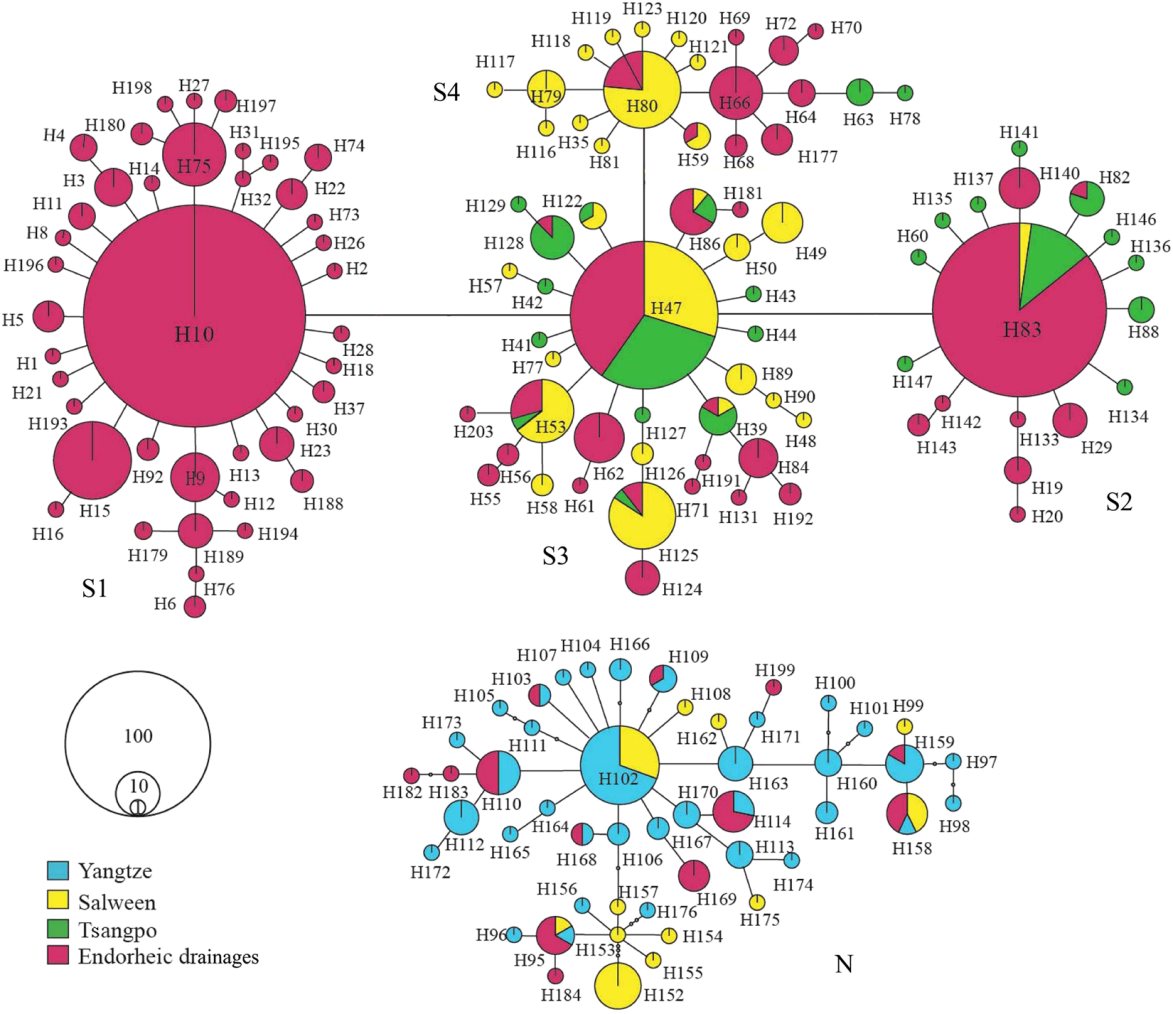
Median-joining network of mitochondrial D-loop haplotypes for the five taxa of schizothoracine fish in this study. Filled circles correspond to unique haplotypes, and their sizes are proportional to haplotype frequency. The five parts of the network correspond to one lineage and four sublineages. The four colours correspond to the four main drainages in our study area (here we treat all endorheic drainages as one). Small open dots indicate missing intermediate haplotypes.

Using sequencing, we obtained 839 bp aligned sites of D-loop sequences, including 81 variable sites, 47 of which were parsimony-informative, and 163 haplotypes. Three common haplotypes (Hap10, Hap47 and Hap83) shared by 455 individuals were identified. The most common haplotype Hap10 (shared by 220 individuals) was private to endorheic populations. In total, 20 haplotypes were shared across drainages (Fig. 2). Overall, both haplotype diversity (h) and nucleotide diversity (π) were high across all populations, with average values of 0.7445 ± 0.17416 and 0.0045 ± 0.0041 (mean ± s.d.), respectively. The populations of three exorheic rivers (NL, LR, ATC, YuC, DC and TH) showed higher π and h than endorheic populations. The three highest h sites (ATC, DC and TH) were located at the transition zone between exorheic and endorheic drainages (Table 2). All ﻿the haplotypes were submitted to GenBank with accession numbers MF685038-MF685200.

**Table 2:** Summary of genetic diversity, neutrality and mismatch analyses of each population.

Site	D-loop									SSR		
	N	h	π	Fu’s *Fs*	Tajiama’s *D*	SSD (P)	Ri	Tau	Growth began, kya (95% CI)	Ai	H_O_	H_E_
TT1	16	0.656	0.0015	−1.80622	−1.32888	0.00216(0.84)	0.03146	2.14	115.3(195.1–63.2)	2.60	0.643	0.850
TT2	17	0.844	0.0061	**−2.62032**	−0.31492	0.00151(0.18)	0.04900	1.79		2.39	0.591	0.855
SC	15	0.889	0.0124	**−5.68972**	−0.7133	0.01536(0.10)	0.05168	2.88		2.66	0.682	0.882
BT	19	0.821	0.003	**−4.26003**	**−1.95308**	0.01527(0.12)	0.07584	2.26		2.49	0.632	0.871
PC	13	0.586	0.0024	**−4.17854**	**−2.19562**	0.03185(0.13)	0.08640	0.67	21.8(28.3–17.6)	2.44	0.615	0.873
NaC	22	0.771	0.0025	**−3.61855**	−1.06759	0.0133(0.46)	0.05150	3.23	77.2(148.6–47.8)	2.38	0.589	0.858
MC	12	0.676	0.0022	−2.28126	−0.79545	0.04338(0.22)	0.16480	3.29	58.7(74.3–18.3)	2.54	0.652	0.851
KC	26	0.872	0.0134	−0.38542	1.28883	**0.04091(0.04)**	0.01300	6.52	43.8(77.1–10.5)	2.45	0.612	0.843
YC	10	0.459	0.0063	−0.80531	−1.21009	0.01708(0.10)	0.17679	0.59	36.2(69.5–19.8)	2.49	0.632	0.848
GC	3	0.465	0.0009	3.41636	−0.70289	**0.26121(0.00)**	0.21524	0.48	—	2.44	0.615	0.773
NC1	10	0.758	0.0025	**−8.71945**	**−1.31482**	0.02172(0.27)	0.06494	3.23	55.8(95.6–21.8)	2.48	0.615	0.843
NC2	12	0.792	0.0021	**−4.7548**	−1.0241	0.02341(0.24)	0.05471	3.06		2.45	0.608	0.832
BC	5	0.334	0.0011	−1.76001	−1.33196	0.03082(0.22)	0.35734	2.0	42.9(46.3–12.8)	2.39	0.596	0.811
ML	16	0.722	0.0016	**−10.3530**	−1.32417	0.00891(0.08)	0.09448	3.75	101.3(133.5–70.5)	2.42	0.604	0.863
LR	15	0.873	0.002	**−10.1344**	−1.69583	0.00245(0.54)	0.06768	3.62		2.52	0.613	0.881
ATC	32	0.913	0.0129	**−3.75926**	1.01608	0.04675(0.18)	0.02066	4.88	137.2(252.6–48.7)	2.46	0.605	0.865
YuC	13	0.878	0.0032	−2.7032	0.34479	0.00064(0.33)	0.03037	3.42		2.38	0.601	0.849
DC	18	0.930	0.0032	**−10.3569**	−1.02339	0.00064(0.89)	0.03386	2.69	130.7(188.6–89.1)	2.41	0.582	0.893
TH	22	0.899	0.0032	**−13.4682**	−1.17882	0.00294(0.55)	0.02884	2.91		2.31	0.567	0.888

N, the number of D-loop haplotypes; h, haplotype diversity; π, nucleotide diversity; Ai, the average number of alleles per individual at a locus; H_O_, observed heterozygosity; H_E_, expected heterozygosity; SSD and Ri, Sum of squares deviations and Raggedness index of mismatch distribution; Tajiama’s *D* and Fu’s *Fs* are statistics of neutrality tests; Significant values (P ≤ 0.05) are indicated in bold. When estimate the growth began data, we treat each river as one site, and Siling Co and directly connected rivers as one site.

For SSR analyses, 671 specimens were used (Table 1). All 12 SSR loci showed a high level of polymorphism, and 318 alleles were amplified. The number of alleles per locus ranged from 20 to 39 (Table S1, Supplementary information), with an average of 2.46 ± 0.08 alleles per individual at each locus (Ai). The expected heterozygosity (H_E_) was high for most populations (0.854 ± 0.028), while the observed heterozygosity (H_O_) was lower (0.6134 ± 0.026). For all endorheic populations, Siling Co showed the highest h, H_O_ and H_E_ (Table 2).

### Phylogenetic analyses and genetic structure

Phylogenetic analyses of D-loop haplotypes were conducted using Bayesian inferences (BI) and maximum likelihood (ML). The BI and ML methods obtained similar topologies; therefore, we only present the BI tree (Fig. S1, Supplementary information). Two well-supported lineages, North (N) and South (S), were identified, separated by the intervening Tanggula Mountains. Lineage N was simple, mainly comprising all upper Yangtze River haplotypes, while lineage S was complex, mainly comprising four obviously sublineages (S1, S2, S3 and S4) in spite of low bootstrap values. Congruent with the phylogenetic tree, the haplotype network (median-joining) analysis showed two isolated networks that corresponded to the two lineages. The lineage corresponding to lineage S was composed of four clusters, with cluster S3 (composed of all three drainages) at the centre and connected to cluster S1 (composed of only endorheic drainage), cluster S2 (mainly composed of endorheic and Tsangpo drainages) and cluster S4 (mainly composed of endorheic and Salween drainages). Each cluster was arrayed in a star-like structure around a central haplotype, which was regarded as the ancestral haplotype (Hap10, Hap47, Hap80, Hap83 and Hap102, Fig. 2).

In accordance with the phylogenetic analyses, spatial genetic analyses of mtDNA haplotypes revealed high values of uncorrected pairwise distance (Fst) between the upper Yangtze River (UY) and other populations (0.849 ± 0.09), suggesting that two defined groups were significantly differentiated. Fst values among populations south of the Tanggula Mountains were low (0.185 ± 0.117). Most of the Fst values among drainages (97%) were significantly higher than zero, suggesting a distinct genetic structure among drainages (Table S3, Supplementary information). An analysis of molecular variance (AMOVA) revealed that a majority of D-loop sequence variation was explained by drainage (59.29%), whereas habitat (different sites within drainage) explained only 8.75%. In addition, a significant amount of variation (31.95%) existed within populations (Table 3).

**Table 3:** Analysis of molecular variance (AMOVA) of D-loop sequences.

Source of variation	d.f.	Sum of squares	Variance components	% variation
Among drainages	7	1383.504	2.64554 Va	59.29
Among sites within drainages	11	214.091	0.39043 Vb	8.75
Within sites	820	1161.631	1.4.2562 Vc	31.95
Total	838	2759.226	4.46203	100

**Figure 3:**
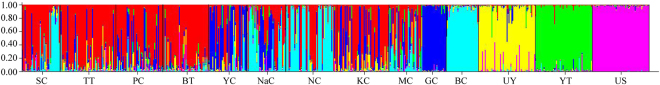
Population structure estimated from 12 nuclear microsatellite loci using STRUCTURE at K = 6. Each thin vertical line corresponds to a single individual, and the six colours represent the six genetic population clusters. Here, we treat each river as a single site. YT, Yarlung Tsangpo; US, upper Salween; UY, upper Yangtze; whole names of other codes are listed in Table 1.

The SSR marker analyses also revealed clear genetic differentiation among drainages. When conducting the STRUCTURE analysis, LnP (D) showed no peak and increased gradually up to K = 14, but the increase slowed after K = 6. In addition, ∆K declined severely from K = 2 to K = 4 and then showed a clear peak at K = 6 (data not shown). Therefore, K = 6 was considered as the most likely number of clusters. At K = 6, the drainages were separated from each other, except for Pongok Co (PC) and Nam Co (NC), which comprised a complex cluster with populations of Siling Co drainage (Fig. 3). The SSR analyses revealed a lower Fst than did the D-loop analyses. Based on SSR markers, the mean Fst between UY and other populations was 0.144 (±0.007) and among populations south of the Tanggula Mountains was 0.037 (±0.002). Significant Fst values occurred in 56 of 91 tests, ranging from 0.024 to 0.185 (Table S3, Supplementary information).

### Demographic history

**Figure 4:**
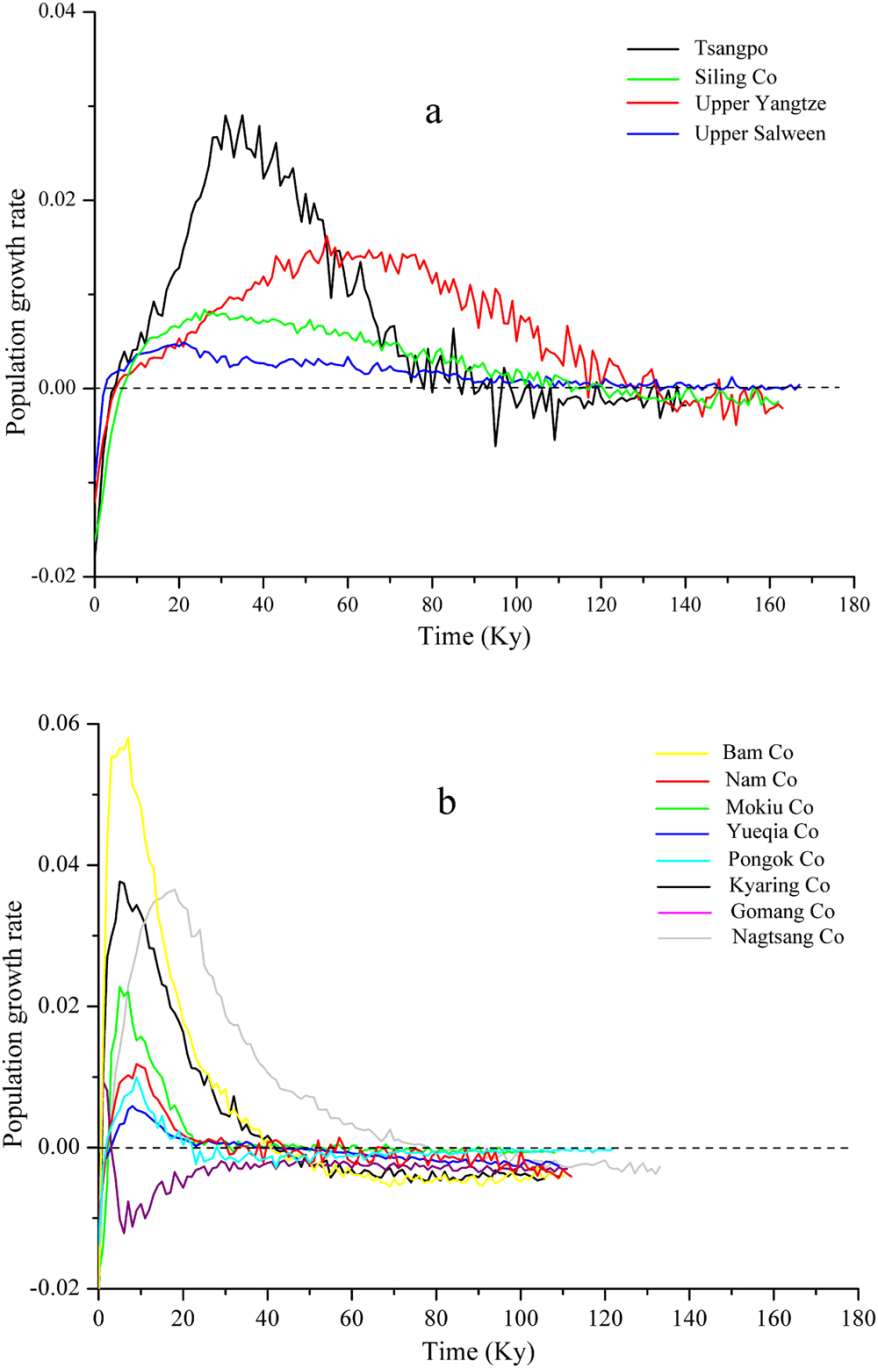
Variation of the instantaneous population growth rate reconstructed by Bayesian skyline plot based on D-loop sequences. The populations of three exorheic rivers and Siling Co (**a**) showed longer expansion history than other endorheic lakes (**b**). The x-axis is the timescale before present, and the y-axis is the estimated population instantaneous growth rate (per millennium) calculated by the equation r = (N_t2_ − N_t1_)/ N_t1_.

For neutrality tests, non-significant negative values of Tajima’s *D* were obtained for most populations, and significantly negative values of Fu’s *Fs* were obtained for more than half populations (Table 2), indicating departure from neutrality. Compared with Tajima’s D, Fu’s Fs is more sensitive in detecting population expansion^28^. Commonly, partial population reduction, population subdivision, secondary contact among lineages or a recent bottleneck can cause discrepancy in detecting population expansion with these two neutrality test statistics^29^. The non-significant negative values of Fu’s *Fs* occurred in population of small lakes, which are easy effected by accidental events. Mismatch distribution analysis showed similar results. The values of sum of squares deviations (SSD) and the raggedness index (Ri) calculated by goodness-of-fit tests were not significant (*P* > 0.05) for most populations (Table 2). Most populations exhibited an observed unimodal mismatch frequency distribution, except for the Pongok Co and Gomang Co (bimodal), indicating that the model of sudden expansion could not be rejected (Fig. S2, Supplementary information). The historical population size inferred by Bayesian skyline plots (BSP) showed three growth patterns (Fig. 4). Gomang Co has been decreasing since the last interglacial period. The three exorheic rivers (Tsangpo, upper Salween and Yangtze River) and Siling Co showed a similar tendency. They began to grow at approximately 100 kya, corresponding to the last interglacial period (130–25 kya), and continued throughout the LGM. For other endorheic populations (BC, NC, MC, YC, PC, KC, and NaC), the rapid growth period began approximately 20 kya, corresponding to post LGM.

### Species distribution modelling

**Figure 5:**
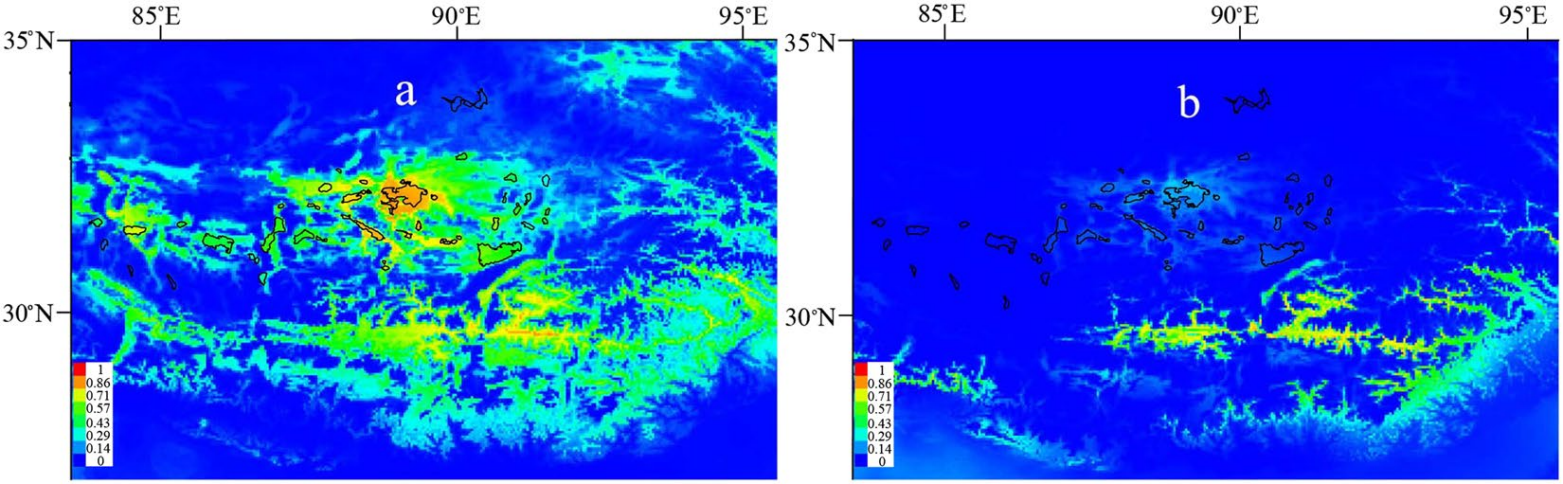
Potential range of the five taxa of schizothoracine fishes on the Qiangtang Plateau in present day (**a**) and during the LGM (**b**). The predicted range during the LGM was based on a community climate system model (CCSM). The colour gradient from blue to red indicates the suitability levels from low to high. The maps were drawn using ArcGIS v10.0 and Adobe Illustrator CS5 v15.1.0.

We used MaxEnt v3.3.3 to predict the potential range of the five taxa in the present and during the LGM based on 97 location records (Table S4). These five taxa have similar ecological requirements and occupied similar habitats and were therefore pooled together in our range prediction analyses. The average testing of the area under the curve (AUC) of 5 replications was 0.881 ± 0.030, implying that our model had good predictive power. The mean temperature of the warmest quarter was estimated as the main limiting environmental variable during the LGM. The predicted present distribution range was consistent with the extant distribution of those five taxa^22^. The community climate system model (CCSM) and the model for interdisciplinary research on climate (MIROC) predicted similar suitable habitat ranges during the LGM; therefore, we only show the result from the CCSM (Fig. 5). During the LGM, suitable habitats on the central QTP were largely reduced and mainly restricted at the south-eastern edge of the QTP. Notably, the area near Siling Co also showed a certain degree of habitat suitability, indicating that schizothoracine fishes may have persisted in this area during the LGM.

## Discussion

The results from the phylogeny and genetic structure analyses of schizothoracine fishes on the central QTP were inconsistent with the hypothesis of “edge refugia”. Commonly, glacial refugia tend to harbour more ancestral haplotypes and higher genetic diversity than recolonized regions, and populations derived from separated refugia show clear genetic differentiation due to drifts^3, 5^. In addition, postglacial expansion would create clines of decreasing genetic diversity along the expansion route, with increasing distance from refugia because of founder effects and bottlenecks^3, 30^. This phenomenon has been widely observed in European biota along a south–north gradient^31^. If the south-eastern and (or) eastern edge of the QTP acted as refugia for fish on the central QTP, the endorheic populations would not have a distinct genetic lineage, and the genetic diversity would have gradually decreased from peripheral populations to the interior. This was not the case in our study.

In our study, phylogenetic analyses and network analyses showed that the sublineage S1 of D-loop haplotypes was generally private to endorheic populations (Figs 1 and 2). Secondly, although exorheic populations showed high genetic diversity, there was no clear cline of genetic diversity decrease from the periphery to the interior. Instead, for all endorheic lakes, the most interior Siling Co harboured the highest genetic diversity, and we detected a clear tendency of decreasing genetic diversity from Siling Co to the outer lakes, for example, from Siling Co to Mokiu Co and to Yueqia Co (Table 2). Thirdly, four ancestral haplotypes (Hap10, Hap47, Hap80 and Hap83) were uncovered in lineage S using network analysis (Fig. 2). All four ancestral haplotypes were detected in the Siling Co area (Table S2, Supplementary information). It is unlikely that these ancestral haplotypes arrived by postglacial dispersal from exorheic rivers, as only two of them were found in Yarlung Tsangpo (Hap47 and Hap83) and three in Salween (Hap47, Hap80 and Hap83, Fig. 2). A logical explanation is that the ancestral haplotypes existed in the glacial refugia of this region over a long period and survived the LGM *in situ*. The above results suggest that schizothoracine fishes on the Qiangtang Plateau survived the LGM in local refugia and that Siling Co, the largest lake in Tibet (2 200 km^2^), which is lower than most surrounding lakes^32^ and is likely a cryptic glacial refugia for local fishes.

The results from demographic history reconstruction, SDM and previous geological studies support our speculation. Glacial refugia provide protection from glaciations; therefore, populations in refugia would have a longer demographic history than would postglacially expanded populations^3^. The demographic history reconstruction revealed that the most recent population growth of three exorheic rivers and Siling Co began approximately 100 kya, corresponding to the last interglacial period (130–25 kya). For most other endorheic populations, fast growth began approximately 20 kya, corresponding to post-LGM (Fig. 4). The Nagtsang Co population also showed earlier expansion than most endorheic populations, possibly because it is located just beside the Siling Co and is well connected. A similar demographic history was also suggested by He *et al*.^33^. The SDM also demonstrated that while most suitable habitats disappeared on the Qiangtang Plateau during the LGM, the Siling Co area showed a certain degree of suitability for those five taxa to live (Fig. 5). In addition, the geological studies suggest that the equilibrium line around the Siling Co basin (approximately 5600 m asl at present) descended by approximately 300 m during the LGM^6, 34^; therefore, Siling Co (4540 m asl) was approximately 800 m lower than the equilibrium line during the LGM. In our sampling sites, Yueqia Co (YC, 4810 m asl) was approximately 800 m lower than the equilibrium line. Now that schizothoracine fishes can currently complete their life history in Yueqia Co, they were likely able to complete their life history in Siling Co during the LGM. Using phylogeographic analyses, Wang *et al*.^35^ and Wang *et al*.^36^ discovered the highest microrefugia (just above 4000 m asl) ever reported. With an elevation of 4540 m asl, Siling Co is the highest glacial microrefugia known.

Phylogram from D-loop haplotypes revealed two well-supported lineages (lineages N and S) separated by the high intervening Tanggula Mountains. Lineage S was further divided into four main sublineages. Freshwater fish have restrictive aquatic requirements for dispersal. However, we noticed that the bootstrap values of the four sublineages are low (Fig. S1, Supplementary information). Except for sublineage S1, which was generally private to endorheic drainages, each of the other three sublineages (S2, S3 and S4) was composed of individuals from at least two drainages, and many haplotypes were shared by at least two drainages (Figs 1 and 2). In addition, the Fst values among the drainages south of Tanggula Mountains were low (Table S3, Supplementary information). These results indicated a certain degree of recent migration among drainages^37^. In total, four or five glaciations have occurred on the QTP since the Pleistocene, the last being the LGM^7, 38^. During the warm and wet interglacial periods, increased rainfall and melting water expanded the lake’s area and generated several temporary channels among drainages^39^. For example, during the last interglacial period, the QTP experienced a “high lake level period” (40–25 kya), in which the temperature was 2–4 °C higher and precipitation was 40–100% higher than today^40^. During that period, many presently isolated lakes, such as Nam Co, Siling Co and Pangkog Co, were expanded several times and connected with each other. In addition, the endorheic and exorheic drainages were also connected at certain times by temporary watercourses^40–42^. These temporary connections propelled gene flow among drainages, reducing their phylogenetic and genetic differentiation. They also served as migration routes for glacial retreat and postglacial dispersion.

The combined use of mtDNA sequences and nuclear SSR gives a more precise view of species evolutionary history and thus increases the performance of phylogenetic studies^43^. Compared with mtDNA sequences, SSR markers presented higher variability and faster mutation rate and provided more information to detect recent divergence^44^. In this study, most results from mtDNA data are consistent with those from nuclear microsatellites, except for the Bam Co and Gomang Co populations. In mtDNA analyses, haplotypes from Bam Co and Gomang Co group with those from other endorheic populations (Fig. 1), but the nuclear microsatellite analysis showed that they have a relatively independent population structure (Fig. 3). The specific geographic feature of those two lakes may contribute to this discordance. Gomang Co is located in a valley, with a small basin area but a high watershed with other lakes (60 m above its present level). For Bam Co, the watershed is much lower in the east than in other directions. During the “high lake level period”, when many endorheic lakes were connected, Gomang Co was still isolated from other lakes, and Bam Co was connected with eastern lakes rather than the Siling Co lake group in the west^45^. These recent divergence could have been detected by SSR analyses but not mtDNA analyses.

In summary, our study provides a comprehensive evaluation of how Pleistocene climatic oscillations have influenced the genetic variation of schizothoracine fishes on the central QTP and gives one example of such processes for the QTP freshwater fishes. Phylogeographic structure and SDM support the scenario of multiple glacial refugia on the plateau platform during the LGM and suggest Siling Co (4540 m asl) to be a cryptic microrefugia for schizothoracine fish on the central QTP that survived the LGM. Our study also illustrates how postglacial lake expansion has influenced the genetic structure among the drainages of those species, especially for the endorheic drainages.

## Materials and Methods

### Ethics statement

The methods involving animals in this study were conducted in accordance with the Laboratory Animal Management Principles of China. All experimental protocols were approved by the Ethics Committee of the Institute of Hydrobiology, Chinese Academy of Sciences.

### Sample collection and DNA extraction

Specimens were sampled with gill nets and electrofishing. Fish identification was according to Fauna Sinica^22^. Measurements were taken immediately after collection. The fin tissue of each specimen was collected and preserved in 95% ethanol. Total genomic DNA was extracted using a standard phenol–chloroform method^46^. DNA concentration was estimated using a NanoDrop 2000 supermicro spectrophotometer (Thermo Fisher Scientific Inc., USA) and adjusted to 50 ng /μL for polymerase chain reaction (PCR).

### MtDNA amplification and genetic diversity analysis

The mtDNA D-loop sequence was amplified using the pair of primers DL (5′-ACCCCTGGCTCCCAAAGC-3′) and DH (5′-ATCTTAGCATCTTCAGTG-3′) in a 60 μL mixture containing 6 μL 10 × PCR buffer (600 μM Tris-HCl, pH 8.3, 3 mM KCl, 90 μM MgCl_2_), dNTPs at 150 μM, forward and reverse primers at 10 pM, 1.0 unit Taq polymerase (TaKaRa) and 50 ng genomic DNA. PCR was performed with a preliminary denaturation at 94 °C for 3 min; followed by 30 cycles of strand denaturation at 94 °C for 40 s, annealing at 55 °C for 50 s and primer extension at 72 °C for 45 s; and a final extension at 72 °C for 10 min. The PCR products were sequenced on an ABI 3730 capillary sequencer from the Sangon Biotech Company (Shanghai, China). Sequences were checked with Bioedit v. 7.2.5^47^ and corrected manually.

The D-loop sequences were initially aligned with the program Clustal × 2.1^48^. All sequences were collapsed into unique haplotypes, and variable sites were counted with DnaSP v5.10^49^. Basic statistics (haplotype number (N), mean number of pairwise differences (p), haplotype (h) and nucleotide (π) diversity) were calculated for each population with Arlequin v 3.5.1^50^.

### MtDNA phylogenetic and population genetic analyses

Phylogenetic analyses of D-loop haplotypes were conducted using BI and ML, by MrBayes v3.2.3^51^ and PhyML 3.0^52^ respectively. Two D-loop sequences of *Gymnocypris przewalskii* and *Gymnocypris eckloni* were designated as the outgroup. The best nucleotide substitution model (GTR + G + I) was selected among 88 alternative models by the Bayesian Information Criteria (BIC) using jModelTest v2.1.4^53^. For Bayesian analyses, two independent runs with four (one cold and three incrementally heated) Markov Chain Monte Carlo (MCMC) were conducted with the GTR + G + I substitution model, run for 20 000 000 generations and 1/1000 sample frequency. Based on convergence diagnostics, the first 4 000 trees (standard deviation of split frequencies <0.01) were discarded as burn-in. Nodal support was calculated using the mean posterior probability (BBP) values of each node of the resulting consensus tree after burn-in. For ML analysis, the node support was assessed using the nonparametric bootstrap method with 1000 replicates. The tree was visualized with FigTree v1.4.2^54^.

A statistical parsimony network of haplotypes was constructed using TCS1.2.1^55^ with a 95% criterion for a parsimonious connection. Excessive connections in the network were removed based on coalescent theory^56^. Genetic differentiation among populations was assessed by pairwise genetic distance (Fst) values. In this section, we treated every river as a single site. The genetic structure within and among drainages was further estimated by AMOVA. Populations within one drainage were pooled into one group. Both analyses were implemented in Arlequin v3.5.1^50^.

### MtDNA demographic history analyses

To assess the historical changes in effective population size, we used multiple approaches. First, we calculated Tajima’s *D* ^57^ and Fu’s *Fs* ^58^ tests to detect population expansions. The significance of both values was calculated from 10 000 simulated samples using a coalescent algorithm. Second, we performed pairwise mismatch distribution analysis (MDA) to compare theoretical distributions under sudden expansion with observed data. Goodness-of-fit was evaluated by the SSD and Raggedness index (Ri) between observed and expected mismatch distributions. The above two analyses were conducted using Arlequin 3.5.1^50^. Finally, we generated the BSP for each population using a Bayesian MCMC algorithm in BEAST 1.8.2 and BEAUti 1.8.2 to convert the molecular sequences of each lake into the input file^59^. The best nucleotide substitution model was selected by jModelTest 2.1.4., using the Akaike Information Criterion (AIC).

An evolutionary rate of 0.91% per million years for the cyt *b* sequence of the genus *Gymnocypris* has been calibrated by the isolation between the upper reaches of the Yellow River and Qinghai Lake, which is a reliable geological event^25^. We calculated the divergence rate of cyt b and D-loop sequences between *H. microcephalus* in Tuotuo He and *Schizopygopsis pylzovi* in the Yellow River using MEGA 6.0^60^. Based on the observed divergence rate of 1.98% for cyt b and 2.32% for the D-loop, we estimated an evolutionary rate of 1.07% per million years for the D-loop sequence. We used a strict clock model and four independent runs of 200 million generations, sampling every 2 000 runs. Tracer 1.6 was used to confirm stationary and convergence, determine the burn-in value (10%) and create the BSP^61^. All runs were combined using LogCombiner 1.8.2 and created a consensus tree using TreeAnnotator 1.8.2, from the BEAST package.

### SSR genotyping and population structure analyses

In total, 33 published SSR primer pairs of *Schizopygopsis younghusbandi* ^62, 63^ were used to screen polymorphisms in 40 individuals from four populations (SC, LR, TH and ATC). PCR was performed as previously described, except for some variations in annealing temperature. The amplified fragments were separated on a 12% non-denaturing polyacrylamide gel. We chose 12 pairs of highly polymorphic SSR primers that amplified reliably across four populations for further genetic analyses (Table S1). Each forward primer for the 12 pairs was labelled with fluorescent dyes (FAM or HEX). PCR products were visualized on an ABI 3730xl DNA Analyser (Beijing Tianyi Huiyuan Bioscience & Technology Corporation). Every individual presented up to 4 alleles, indicating that the samples were tetraploids. Therefore, the genotypes of samples were assessed by the fluorescence height of the alleles according to the Microsatellite DNA Allele Counting Peak Ratios (MAC-PR) method^64^.

The number of alleles per locus (Na), allelic richness (Ai) and observed (H_O_) and expected heterozygosity (H_E_) were calculated using the program AUTOTET^65^. Fst values among populations were estimated by POLYSAT^66^. The population genetic structure was assessed by a Bayesian model-based method in STRUCTURE 2.3.4^67^. We chose the admixture model for the ancestry of individuals and the correlated model for allele frequencies. The length of burn-in was set to 10 000, with 100 000 MCMC replication runs after burn-in. To avoid bias, we performed the program from K = 1 to 14, with 30 iterations at each value of K. The optimum value of clusters (K) was determined by comparison of mean log probability Ln P (D) and by calculating the ∆K value for each K^68^.

### Species distribution modelling

We used SDM to predict the potential range of these five taxa in the present and during the LGM. Models were generated with MaxEnt v3.3.3^69^, a program for maximum entropy modelling of the geographical distributions of species. Overall, 97 location records were collected, including 19 from the present study and 78 from our previous survey from 2000 to 2015 (Table S4). We downloaded 19 commonly used bioclim-variables from WorldClim (http://www.worldclim.org), with a spatial resolution of 2.5 arc-minutes. The altitude data for these sites were recorded by GPS. Highly correlated environmental variables were removed using multicollinearity test by ENMtools v1.3^70^, and six bioclim-variables (annual mean temperature, minimum temperature of coldest month, mean temperature of warmest and coldest quarter, annual precipitation and precipitation of wettest quarter) and altitude were ultimately used.

Both the CCSM and MIROC were used to generate the environmental variables for the LGM. Those data layers were also downloaded from WorldClim. We chose the climatic-topographic models (climate model) for both time periods and set the convergence threshold and number of maximum iterations at default values of 0.00001and 500 respectively. We assigned 75% of the occurrence point as training data and the remaining 25% as testing data. The number of maximum iterations and convergence threshold were set at default values of 500 and 0.00001, respectively. We ran five fold cross-validation for each model and produced a single map per time period using the logistic output format, with suitability values ranging from 0 to 1^67^. Model evaluation was based on the AUC^71^.

## Electronic supplementary material

Supplementary information
