# Supplementary material for: Phylogeographic studies of schizothoracine fishes on the central Qinghai-Tibet Plateau reveal the highest known glacial microrefugia
Source: Sci Rep. 2017 Sep 8;7:10983. doi: 10.1038/s41598-017-11198-w (PMC5591315; doi:10.1038/s41598-017-11198-w)
Supplement: Supplementary file 1 — Supplementary information [file 41598_2017_11198_MOESM1_ESM.doc]

**Supplementary information:**

**Phylogeographic studies of schizothoracine fishes on the central Qinghai-Tibet Plateau reveal the highest known glacial microrefugia**

Yangyang Liang^1, 3^*, Dekui He^1,2^, Yintao Jia^1^, Heying Sun^1, 3^ & Yifeng Chen^1^

^1^ The Key Laboratory of Aquatic Biodiversity and Conservation of Chinese Academy of Sciences, Institute of Hydrobiology, Chinese Academy of Sciences, Wuhan, 430072, China.

^2^Southeast Asia Biodiversity Research Institute, Chinese Academy of Sciences, Menglun, Yunnan 666303, China.

^3^ University of Chinese Academy of Sciences, Beijing, 100049, China.

*Corresponding author.

Corresponding author E-mail: liangyy10214@126.com

**Table S1** Characteristics of the 12 SSR primers used in this study

| Marker | Primer sequence(5’- 3’) | Na | Repeat unit | Ta (^o^C) |
| --- | --- | --- | --- | --- |
| C44 | F-GACCTGAACACAGATCAATTGC  R-CCACAACAAAGCATAGCATAGG | 28 | TG | 57 |
| F02 | F-TGGTTCAGGGTCACATATATGG  R-TTCAGCTTTGCACATGCCTT | 21 | AG | 53 |
| F10 | F-ATTTCTCCTGGAGCCAACACAT  R-CAAGACTCAGCCATTACACACA | 20 | AG | 55 |
| F36 | F-TAAATGCAGGCTTACCAACCAG  R-AAAGCCAGCTCAGACTGTTACA | 26 | AC | 57 |
| H52 | F-TGTGGAGCAGCTGTATGACTGT  R-CAAACACGAACGCTGTTTCC | 22 | CA | 55 |
| G57 | F-CGTCCTGCTGTAATCTGCCTTA  R-AATGCCATCACCAGAGCTTCAT | 27 | AG | 62 |
| K19 | F-GATCTCTCCATTCTGTGTAAG  R-AGTTTGTGGTTATGCTCCT | 22 | TAGA | 55 |
| K21 | F-TCCGCTTCACGATTGACTAA  R-ACCCCCATCTCTGCCATT | 39 | ATCT | 51 |
| K22 | F-TTTTAATCCACAGAGATGCC  R-ATCATTCAAAGGTCACTCGT | 27 | TCAT | 56 |
| K26 | F-AGGTTCCTCTTTGTGTTTG  R-CTTCTGCCTCGTTCTGTT | 25 | AGAT | 59 |
| K27 | F-ATCATTCAAAGGTCACTCGT  R-TCCACAGAGATGCCAAAG | 29 | TAGA | 58 |
| K30 | F-CCAAAGCAAATTCTGTTCTT  R-CTCCCTTGTCTGACCTCC | 32 | GATA | 51 |

Na, number of alleles; T_a_, annealing temperature.

**Table S2** D-loop haplotypes detected in each site

| Site | Haplotypes |
| --- | --- |
| TT1 | H9, H10, H19, H23, H47, H56, H66, H75, H83, H86, H158, H169, H177, H179, H181, H184 |
| TT2 | H3, H4, H10, H15, H31, H37, H47, H73, H74, H75, H76, H83, H84, H95, H110,H182, H188 |
| SC | H3, H10, H11, H12, H15, H20, H26, H29, H62, H64, H66, H80, H83 ,H140, H189 |
| BT | H3, H6, H9, H10, H15, H23, H47, H55, H56, H64, H66, H80, H83, H84, H140, H142, H143, H169, H203 |
| PC | H2, H5, H10, H14, H19, H22, H47, H62, H66, H70, H72, H83, H114 |
| NaC | H8, H9, H10, H11,H19, H37, H47,H66, H68, H71, H72, H75, H83, H84, H92, H180, H188, H189, H191, H192, H193, H194 |
| MC | H1, H4, H9, H10, H23, H39, H47, H69, H83, H124, H131, H133 |
| KC | H10, H11,H13,H16, H18, H28, H32, H47, H53, H61, H75, H83, H86, H95, H103, H109, H110, H114, H158, H168, H169, H183, H195, H196, H197, H198 |
| YC | H10, H29, H47, H75, H83, H95, H114, H159, H197, H199 |
| GC | H10, H15, H125 |
| NC1 | H10, H30, H47, H53, H59, H62, H66, H82, H83, H84 |
| NC2 | H10, H15, H21, H29, H47, H53, H84, H66, H83, H124, H125, H128 |
| BM | H10, H22, H27, H83, H143 |
| NL | H39, H42, H47, H63, H78, H82, H83, H86, H60, H122, H128, H129, H135, H136, H137, H146 |
| LR | H39, H41, H43, H44, H47, H53, H82, H83, H88, H125, H127, H128, H134, H141, H147 |
| ATC | H35, H39, H47, H48, H53, H59, H77, H79, H80, H81, H86, H89, H90, H95, H99, H102, H108, H117, H119, H120, H121, H122, H123, H125, H152, H153, H154, H155, H157, H158, H162, H175 |
| YuC | H47, H49, H50, H53, H57, H58, H80, H83, H89, H116, H118, H125, H126 |
| DC | H102, H112, H114, H156, H158, H159, H160, H161, H163, H164, H165, H166, H167, H168, H170, H171, H173, H176 |
| TH | H95, H96, H97, H98, H100, H101, H102, H103, H104, H105, H106, H107, H109, H110, H111, H112, H113, H114, H160, H163, H172, H174 |

**Table S3** Standardized Fst values between all pairs of sites from mtDNA (below the diagonal) and SSR markers (above the diagonal).

|  | SC | TT | BT | NaC | KC | MC | YC | PC | NC | GC | BC | YT | US | UY |
| --- | --- | --- | --- | --- | --- | --- | --- | --- | --- | --- | --- | --- | --- | --- |
| SC |  | 0.018 | 0.010 | 0.013 | 0.024 | 0.019 | 0.014 | 0.014 | 0.018 | **0.031** | **0.035** | **0.049** | **0.047** | **0.121** |
| TT | 0.022 |  | 0.019 | 0.027 | **0.031** | 0.024 | 0.025 | 0.015 | **0.033** | **0.036** | **0.041** | **0.057** | **0.063** | **0.107** |
| BT | 0.044 | 0.057 |  | 0.018 | 0.024 | 0.020 | 0.015 | 0.016 | 0.024 | **0.036** | **0.038** | **0.045** | **0.047** | **0.139** |
| NaC | 0.019 | 0.031 | 0.021 |  | **0.037** | 0.027 | **0.028** | **0.028** | 0.026 | **0.037** | **0.038** | **0.059** | **0.049** | **0.131** |
| KC | **0.145** | **0.181** | **0.190** | **0.185** |  | **0.036** | **0.029** | 0.022 | **0.048** | **0.041** | **0.055** | **0.073** | **0.070** | **0.098** |
| MC | **0.129** | **0.137** | 0.039 | 0.049 | **0.195** |  | 0.026 | 0.015 | **0.037** | **0.034** | **0.044** | **0.066** | **0.056** | **0.149** |
| YC | 0.044 | 0.051 | **0.078** | **0.095** | **0.155** | **0.149** |  | 0.016 | **0.031** | **0.031** | **0.045** | **0.069** | **0.065** | **0.145** |
| PC | **0.071** | **0.061** | **0.061** | **0.089** | **0.194** | **0.194** | **0.101** |  | 0.024 | **0.024** | **0.035** | **0.042** | **0.040** | **0.153** |
| NC | **0.068** | **0.151** | **0.082** | 0.042 | **0.185** | **0.091** | **0.067** | 0.032 |  | **0.041** | **0.027** | **0.075** | **0.065** | **0.162** |
| GC | **0.332** | **0.270** | **0.247** | **0.325** | **0.190** | **0.456** | **0.139** | **0.234** | **0.258** |  | **0.049** | **0.071** | **0.069** | **0.168** |
| BC | **0.363** | **0.326** | **0.212** | **0.209** | **0.263** | **0.109** | **0.321** | **0.422** | **0.325** | **0.532** |  | **0.078** | **0.074** | **0.172** |
| YT | **0.220** | **0.228** | **0.221** | **0.234** | **0.240** | **0.217** | **0.225** | **0.283** | **0.221** | **0.378** | **0.305** |  | **0.043** | **0.142** |
| US | **0.216** | **0.185** | **0.114** | **0.138** | **0.212** | **0.089** | **0.234** | **0.286** | **0.176** | **0.552** | **0.225** | **0.208** |  | **0.185** |
| UY | **0.887** | **0.877** | **0.881** | **0.887** | **0.576** | **0.895** | **0.824** | **0.889** | **0.893** | **0.912** | **0.919** | **0.702** | **0.899** |  |

In this table, we treat every river as one site. YT, Yarlung Tsangpo; US, Upper Salween; UY, Upper Yangzte. Significant values are indicated in bold.

**Table S4.** Occurrence information of the five taxa in this study for SDM.

| Longitude (E) | | Latitude (N) | Species | Longitude (E) | Latitude (N) | Species |
| --- | --- | --- | --- | --- | --- | --- |
| 88.6135 | | 31.8002 | *S.* spp. | 88.5923 | 30.5618 | *S.* spp. |
| 88.5744 | | 31.7413 | *S.* spp. | 85.2497 | 30.6525 | *S.* spp. |
| 88.4841 | | 31.8121 | *S.* spp. | 84.6029 | 29.2493 | *S. younghusbandi* |
| 88.7521 | | 31.1790 | *S.* spp. | 88.5194 | 29.3514 | *S. younghusbandi* |
| 89.2167 | | 31.2503 | *S.* spp. | 89.0833 | 29.6833 | *S. younghusbandi* |
| 89.2645 | | 31.8521 | *S.* spp. | 90.9264 | 30.3853 | *S. younghusbandi* |
| 89.3393 | | 31.7749 | *S.* spp. | 91.8842 | 29.9883 | *S. younghusbandi* |
| 89.2849 | | 31.6202 | *S.* spp. | 94.2213 | 29.1233 | *S. younghusbandi* |
| 89.3705 | | 31.5047 | *S.* spp. | 83.5301 | 29.8680 | *S. younghusbandi* |
| 89.5399 | | 31.7236 | *S.* spp. | 84.0670 | 29.8680 | *S. younghusbandi* |
| 89.6539 | | 31.6341 | *S.* spp. | 85.2740 | 29.3138 | *S. younghusbandi* |
| 91.1204 | | 31.2504 | *S.* spp. | 87.5711 | 29.3849 | *S. younghusbandi* |
| 90.7451 | | 31.3151 | *S.* spp. | 87.7020 | 29.2204 | *S. younghusbandi* |
| 92.5562 | | 30.7841 | *S.* spp. | 89.4639 | 29.0374 | *S. younghusbandi* |
| 88.1000 | | 31.2667 | *S.* spp. | 91.3333 | 31.1833 | *S. younghusbandi* |
| 87.8716 | | 31.3872 | *S.* spp. | 91.2650 | 29.8905 | *S. younghusbandi* |
| 88.6167 | | 30.4667 | *S.* spp. | 90.7642 | 29.3368 | *S. younghusbandi* |
| 88.2319 | | 31.8124 | *S.* spp. | 91.0379 | 29.2958 | *S. younghusbandi* |
| 87.9473 | | 31.7044 | *S.* spp. | 90.1995 | 29.3486 | *S. younghusbandi* |
| 87.2771 | | 31.5838 | *S.* spp. | 91.6799 | 29.2619 | *S. younghusbandi* |
| 87.1284 | | 30.8703 | *S.* spp. | 92.8160 | 29.0698 | *S. younghusbandi* |
| 87.0280 | | 31.7727 | *S.* spp. | 93.5825 | 29.1671 | *S. younghusbandi* |
| 86.6915 | | 31.2223 | *S.* spp. | 94.7591 | 29.4751 | *S. younghusbandi* |
| 85.8179 | | 30.8321 | *S.* spp. | 93.8664 | 28.8186 | *S. younghusbandi* |
| 85.6824 | | 30.5562 | *S.* spp. | 83.3917 | 30.3167 | *S. younghusbandi* |
| 85.3963 | | 31.0276 | *S.* spp. | 84.1848 | 29.5744 | *S. younghusbandi* |
| 86.4285 | | 31.8035 | *S.* spp. | 86.2890 | 29.5213 | *S. younghusbandi* |
| 89.1333 | | 32.0502 | *S.* spp. | 86.2310 | 29.1998 | *S. younghusbandi* |
| 89.5588 | | 32.0277 | *S.* spp. | 88.9124 | 29.2524 | *S. younghusbandi* |
| 90.7337 | | 32.4515 | *S.* spp. | 92.3667 | 33.86667 | *H. microcephalu* |
| 89.0279 | | 31.8252 | *S.* spp. | 92.6513 | 34.1613 | *H. microcephalu* |
| 89.0946 | | 31.6446 | *S.* spp. | 91.5670 | 34.2772 | *H. microcephalu* |
| 88.7453 | | 30.7967 | *S.* spp. | 92.9106 | 34.0937 | *H. microcephalu* |
| 87.9563 | | 31.3777 | *S.* spp. | 92.4333 | 34.2167 | *H. microcephalu* |
| 84.2064 | | 31.1358 | *S.* spp. | 91.8119 | 34.2006 | *H. microcephalu* |
| 88.5512 | | 31.6365 | *S.* spp. | 90.1648 | 30.4622 | *G. namensis* |
| 88.5741 | | 30.2311 | *S.* spp. | 91.1255 | 30.8368 | *G. namensis* |
| 88.9063 | | 30.5515 | *S.* spp. | 90.2719 | 30.8771 | *G. namensis* |
| 88.7140 | | 31.4455 | *S.* spp. | 90.6936 | 30.7151 | *G. namensis* |
| 89.9217 | | 32.4325 | *S.* spp. | 89.6403 | 30.9302 | *G. namensis* |
| 89.7892 | | 31.8606 | *S.* spp. | 89.8660 | 30.9139 | *G. namensis* |
| 89.0051 | | 31.9636 | *S.* spp. | 94.5438 | 31.4607 | *S. thermalis* |
| 88.6833 | 31.6000 | | *S.* spp. | 93.7430 | 31.7668 | *S. thermalis* |
| 89.0049 | 31.0690 | | *S.* spp. | 92.8921 | 31.5651 | *S. thermalis* |
| 90.6541 | 31.2277 | | *S.* spp. | 91.4833 | 31.9333 | *S. thermalis* |
| 87.3878 | 31.5446 | | *S.* spp. | 91.4827 | 31.4912 | *S. thermalis* |
| 90.1982 | 32.2243 | | *S.* spp. | 91.6599 | 31.6998 | *S. thermalis* |
| 88.5608 | 31.0166 | | *S.* spp. | 92.0270 | 31.4443 | *S. thermalis* |
| 89.0167 | 31.0500 | | *S.* spp. |  |  |  |


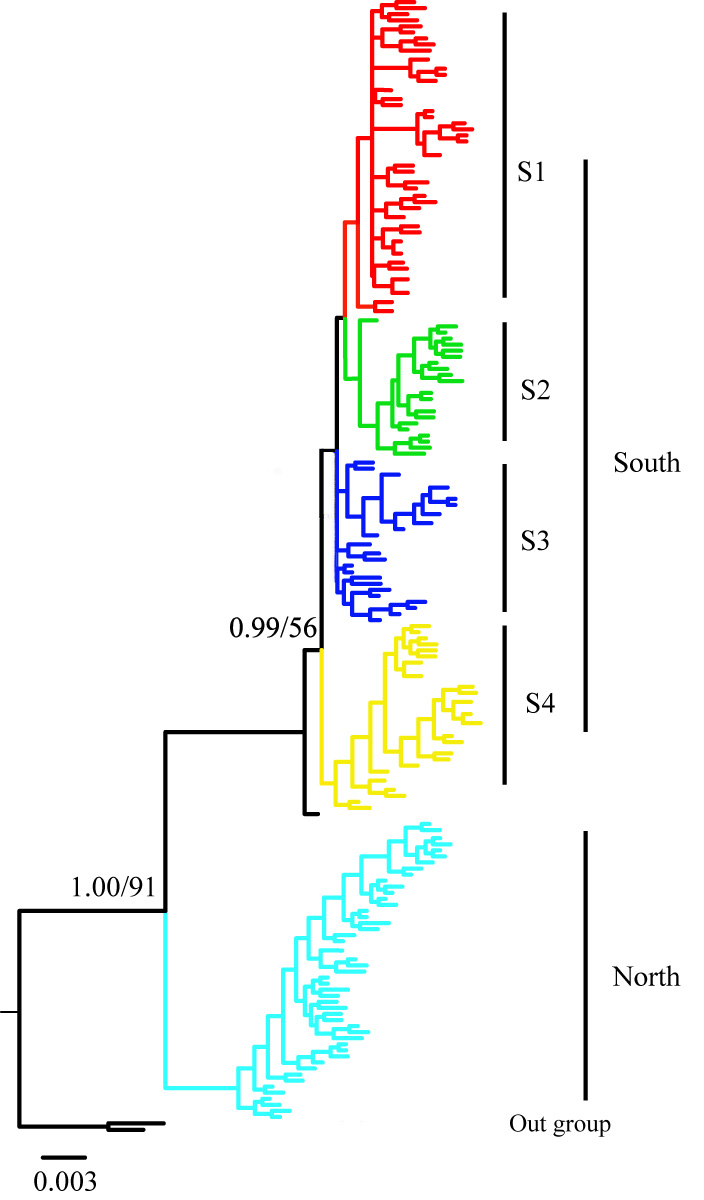


**Figure S1.** Bayesian inferences (BI) tree based on the mitochondrial D-loop haplotypes of the five taxa in this study. Bayesian posterior probabilities/maximum likelihood bootstrap support values are above the branch. Five colors correspond to one lineage and four sublineages. The bootstrap values of other separations were low and therefore don’t have statistical significance, so we don’t present them.


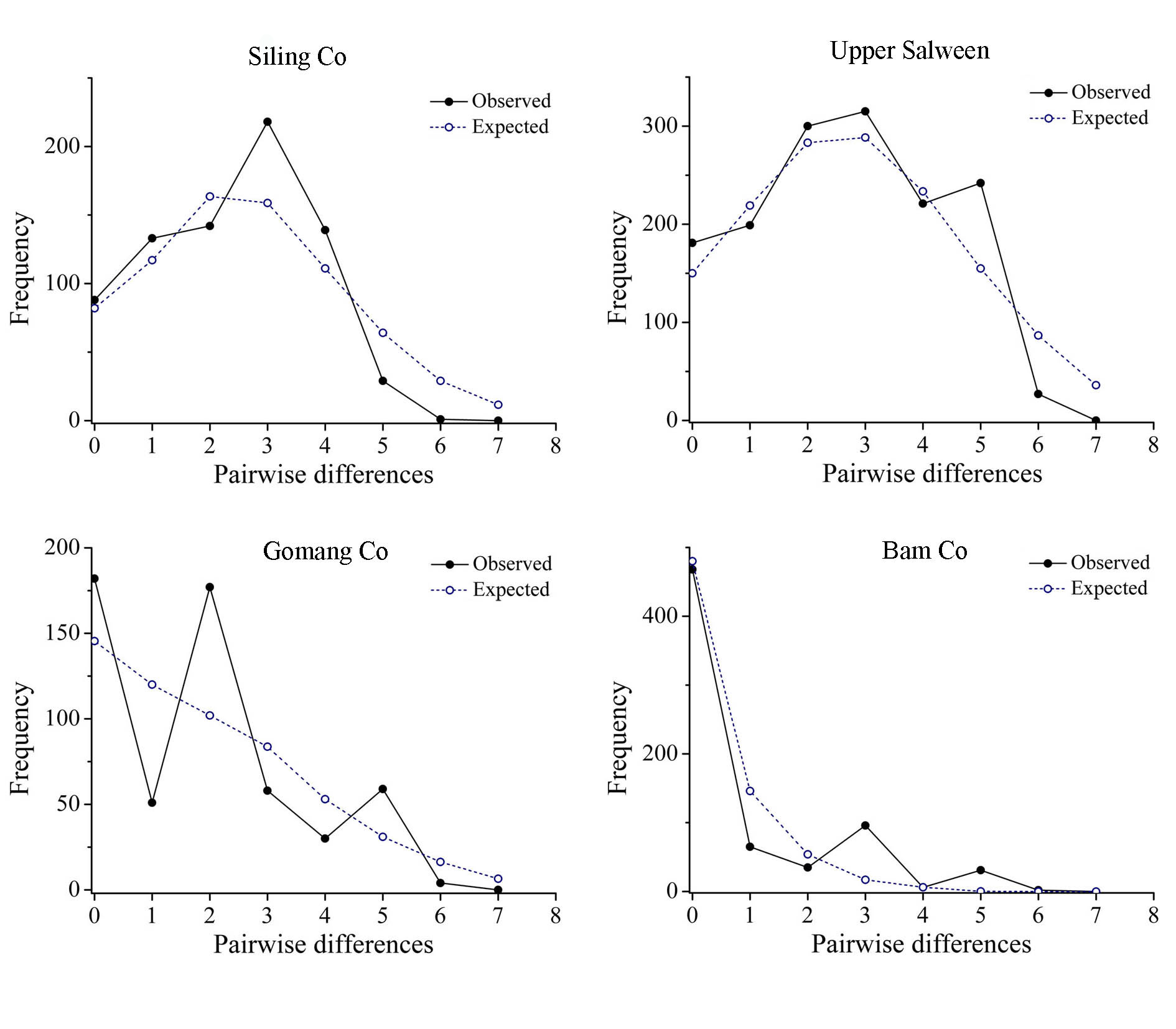


**Figure S2**. Four main patterns of mismatch distributions based on the D-loop sequence data. Black solid lines represent the observed frequency and dash lines represent the expected frequency under the sudden expansion model.
